# Serum glycated albumin as a predictive biomarker for renal involvement of antineutrophil cytoplasmic antibody-associated vasculitis in non-diabetic patients

**DOI:** 10.1186/s12882-022-02913-5

**Published:** 2022-08-18

**Authors:** Pil Gyu Park, Jung Yoon Pyo, Sung Soo Ahn, Jason Jungsik Song, Yong-Beom Park, Sang-Won Lee

**Affiliations:** 1 Division of Rheumatology, Department of Internal Medicine, Yonsei University College of Medicine, 50-1 Yonsei-ro, Seodaemun-gu, Seoul, 03722 Republic of Korea; 2 Institute for Immunology and Immunological Diseases, Yonsei University College of Medicine, Seoul, Republic of Korea

**Keywords:** Glycated albumin, Nephropathy, End-stage renal disease, Antineutrophil cytoplasmic antibody

## Abstract

**Background:**

Glycated albumin (GA) is known to reflect the current inflammatory burden in non-diabetes mellitus (DM) patients. In this study, we investigated whether GA at diagnosis could reflect the cross-sectional activity and predict poor outcomes during follow-up in non-DM patients with antineutrophil cytoplasmic antibody (ANCA)-associated vasculitis (AAV).

**Methods:**

The medical records of 118 immunosuppressive drug-naïve AAV patients were retrospectively reviewed, and 76 patients who had both GA and glycated haemoglobin (HbA1c) results but not DM were included in this study. Demographic, clinical, and laboratory data at diagnosis were assessed.

**Results:**

The median age of AAV patients was 61 years, and 31 patients were male. GA was positively correlated with five-factor score (*r* = 0.282), Birmingham vasculitis activity score (BVAS) assigned to renal manifestation (*r* = 0.315), and blood urea nitrogen (*r* = 0.382), whereas negatively correlated with haemoglobin (*r* = -0.345). AAV patients with end-stage renal disease (ESRD) exhibited significantly higher GA than those without ESRD (15.8% vs. 13.6%). When the cut-off of GA at diagnosis for ESRD was set at GA ≥ 14.25%, AAV patients with GA ≥ 14.25% had a significantly higher risk for ESRD development than those without (relative risk 12.040). In addition, AAV patients with GA ≥ 14.25% exhibited significantly lower cumulative ESRD-free survival rates than those without (*P* = 0.020).

**Conclusion:**

In conclusion, GA at diagnosis can reflect the cross-sectional BVAS assigned to renal manifestation of AAV and predict ESRD development during follow-up better than HbA1c or GA/HbA1c in AAV patients.

**Supplementary Information:**

The online version contains supplementary material available at 10.1186/s12882-022-02913-5.

## Background

Glycation is a process where sugars and proteins are cross-linked because of increased blood sugar concentration. Based on these mechanisms, glycated proteins have been widely used to evaluate changes in blood sugar and the efficiency of blood sugar control. The most representative glycated proteins are glycated haemoglobin (HbA1c) and glycated albumin (GA) [1]. HbA1c has been used to monitor changes in blood glucose for a longer time than GA. The half-life of HbA1c is 3 to 4 weeks, and HbA1c may reflect changes in blood glucose levels for the previous 3 to 6 months; in contrast, the half-life of GA is as short as 12 to 21 days, and thus, GA may reflect changes in blood glucose for the previous 3 to 4 weeks [2]. For this reason, the clinical significance of GA in actual clinical practice is gradually increasing.

Meanwhile, once sugars are bound to proteins, conformational changes in proteins may occur, resulting in functional alterations of the proteins in the blood. Functional alteration may result in diseases, and thus, glycated proteins are significant or reasons other than simply observing blood sugar trends [3]. For example, GA promotes the production of pro-inflammatory cytokines and stimulates protein kinase C, leading to systemic complications and the development of renal insufficiency or nephropathy in diabetes mellitus (DM) patients [4–6]. Thus, GA is known to be a predictor of the systemic complications of DM, in particular diabetic nephropathy [7].

Contrarily, GA also has clinical implications as a biomarker that reflects the degree of systemic inflammation. It has been reported that GA plays a role in the process of atherosclerosis, which can result in cardiovascular diseases [8]. GA stimulates the growth of vascular smooth muscle cells and enhances the production of interleukin (IL)-6, a pivotal cytokine associated with atherosclerosis [9]. For this reason, GA has also been reported to be useful in reflecting the inflammatory burden in non-DM patients with cardiovascular diseases. Moreover, in our previous study, we demonstrated that GA could reflect disease activity in non-DM patients with rheumatoid arthritis. Patients with active rheumatoid arthritis exhibited a significantly higher level of GA than those with inactive rheumatoid arthritis [10].

It is reported that GA can initiate and accelerate the production of pro-inflammatory cytokines, tumour necrosis factor-alpha, IL-6, and IL-8 [5, 11]. These pro-inflammatory cytokines are the main molecules involved in the pathogenesis of antineutrophil cytoplasmic antibody (ANCA)-associated vasculitis (AAV) [12, 13]. Thus, it can be hypothesised that GA also participates in the pathogenesis of AAV and can reflect the cross-sectional activity of AAV. However, there have been no studies on investigating the clinical significance of GA in patients with AAV. Hence, in this study, we investigated whether GA at diagnosis could reflect the cross-sectional activity of AAV and predict poor outcomes during follow-up in AAV patients without DM. In addition, we compared the clinical significance of GA with that of other glycated proteins such as HbA1c and GA/HbA1c.

## Methods

### Study population

We reviewed the medical records of 118 immunosuppressive drug-naïve AAV patients enrolled in the prospective Severance Hospital ANCA-associated VasculitidEs (SHAVE) cohort. The SHAVE cohort is a prospective and observational cohort that began in November 2016 and includes patients with microscopic polyangiitis (MPA), granulomatosis with polyangiitis (GPA), and eosinophilic GPA (EGPA). All patients in this study met the following inclusion criteria. First, the diagnosis of AAV was performed at the Department of Internal Medicine, Yonsei University College of Medicine, Severance Hospital. Second, AAV was classified based on both the classification algorithm for AAV and polyarteritis nodosa proposed by the European Medicine Agency in 2007 (the 2007 algorithm) [14] and the revised nomenclature of vasculitides suggested by the Chapel Hill Conference Consensus in 2012 (the 2012 definitions) [15]. Third, the medical records of the study population should include information regarding clinical and laboratory data such as AAV subtype, ANCA type, Birmingham vasculitis activity score (BVAS) and five-factor score (FFS) [16, 17]; clinical manifestation and laboratory results at diagnosis; and poor outcomes during follow-up. The exclusion criteria were as follows: a follow-up duration of less than 3 months; concurrent serious medical conditions including malignancies, infections, and systemic vasculitides other than AAV; and previous exposure to immunosuppressive drugs for the treatment of AAV.

**Fig. 1:**
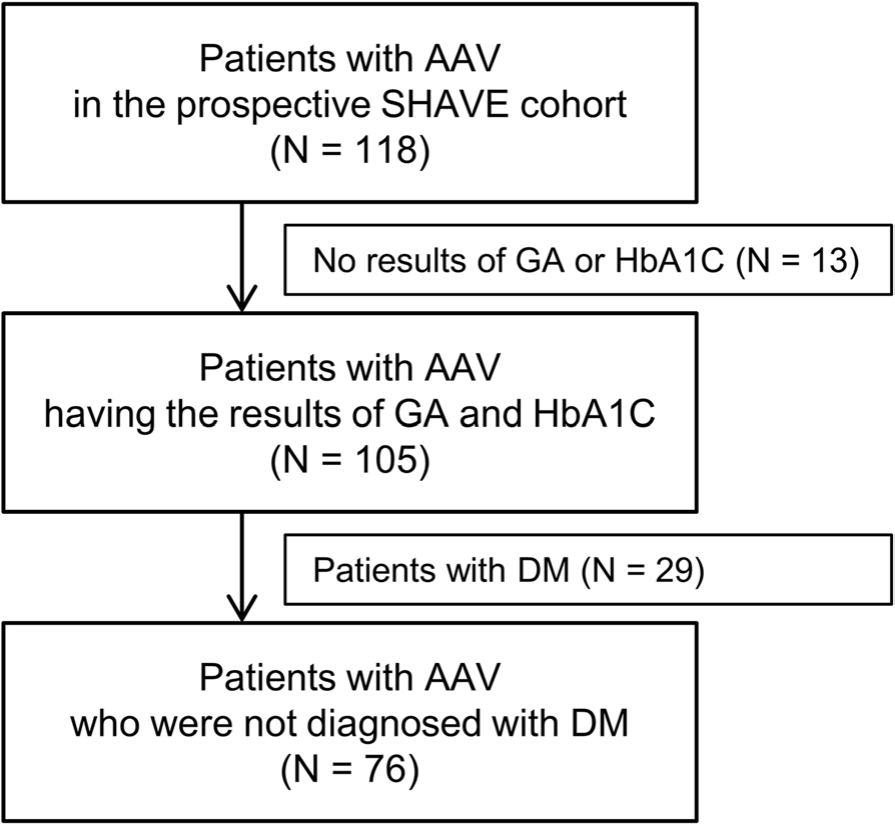
Selection of study subjects. AAV: ANCA-associated vasculitis; ANCA: antineutrophil cytoplasmic antibody; GA: glycated albumin; HbA1c: glycated haemoglobin; DM: diabetes mellitus

Of 118 AAV patients, 13 were excluded because of no results on GA or HbA1c at diagnosis. Of 105 AAV patients with GA and HbA1c results, 29 patients were diagnosed with concurrent DM. They were also excluded to avoid confusion in interpreting the results of this study. Finally, 76 AAV patients who had GA and HbA1c results, and were not diagnosed with DM at the time of AAV diagnosis were included in the present study (Fig. 1). The Institutional Review Board (IRB) of Severance Hospital (Seoul, Korea, IRB No. 4–2020-1071) approved this study. The requirement for written informed consent was waived because of the retrospective design of the study and the use of anonymised patient data.

### Demographic, clinical and laboratory data at diagnosis

Demographic, clinical, and laboratory data at diagnosis were collected as described in Table 1. Immunoassays were used as the primary screening method for ANCA. However, patients with negative for ANCA by an antigen-specific assay but positive for perinuclear (P)-ANCA or cytoplasmic (C)-ANCA by an indirect immunofluorescence assay, were considered to have myeloperoxidase (MPO)-ANCA or proteinase 3 (PR3)-ANCA, especially when AAV was strongly suspected based on clinical and laboratory features [18]. As inflammation-related biomarkers, GA, HbA1c, and GA/HbA1c at diagnosis were measured and recorded.

**Table 1:** Characteristics of AAV patients without DM (*N* = 76)

Variables	Values
**Demographic data at diagnosis**	
Age (years)	61.0 (21.0)
Male sex (N, (%))	31 (40.8)
**AAV subtypes (N, (%)) at diagnosis**	
MPA	42 (55.3)
GPA	18 (23.7)
EGPA	16 (21.1)
**ANCA positivity (N, (%)) at diagnosis**	
MPO-ANCA (or P-ANCA) positive	42 (55.3)
PR3-ANCA (or C-ANCA) positive	9 (11.8)
Both ANCA positive	2 (2.6)
**AAV-specific indices at diagnosis**	
BVAS	6.0 (7.0)
FFS	1.0 (1.0)
**Clinical manifestations at diagnosis (N, (%))**	
General manifestations	14 (18.4)
Cutaneous manifestations	10 (13.2)
Mucous and ocular manifestations	5 (6.6)
Otorhinolaryngologic manifestations	27 (35.5)
Pulmonary manifestations	48 (63.2)
Cardiovascular manifestations	2 (2.6)
Gastrointestinal manifestations	0 (0)
Renal manifestations	41 (53.9)
Nervous systemic manifestations	22 (28.9)
**Acute phase reactants at diagnosis**	
ESR (mm/hr)	16.5 (35.0)
CRP (mg/L)	1.4 (5.3)
**Inflammation-related biomarkers at diagnosis**	
GA (%)	13.9 (2.7)
HbA1c (%)	5.6 (0.7)
GA/HbA1c	2.4 (0.5)
**Laboratory results at diagnosis**	
White blood cell count (/mm^3^)	6,775.0 (4,340.0)
Haemoglobin (g/dL)	12.4 (3.2)
Platelet count (× 1000/mm^3^)	244.0 (99.3)
Blood urea nitrogen (mg/dL)	18.7 (19.0)
Serum creatinine (mg/dL)	0.9 (1.0)
Total protein (g/dL)	6.6 (0.7)
Serum albumin (g/dL)	4.2 (0.6)
Random urine protein/creatinine ratio	0.2 (0.7)
Red blood cells per high power field	0 (10.0)
**Medications (N, (%)) during follow-up**	
Glucocorticoids	74 (97.4)
Cyclophosphamide	49 (64.5)
Rituximab	18 (23.7)
Mycophenolate mofetil	12 (15.8)
Azathioprine	52 (68.4)
Tacrolimus	4 (5.3)
Methotrexate	8 (10.5)
Plasma exchange	10 (13.2)
**Poor outcomes during follow-up**	
All-cause mortality	5 (6.6)
Follow-up duration based on all-cause mortality (months)	28.7 (31.1)
Relapse	23 (30.3)
Follow-up duration based on relapse (months)	18.5 (31.2)
ESRD	8 (10.5)
Follow-up duration based on ESRD (months)	27.0 (31.1)

Values are expressed as a median (interquartile range, IQR) or N (%)

*AAV* ANCA-associated vasculitis, *ANCA* Antineutrophil cytoplasmic antibody, *DM* Diabetes mellitus, *MPA* Microscopic polyangiitis, *GPA* Granulomatosis with polyangiitis, *EGPA* Eosinophilic granulomatosis with polyangiitis, *MPO* Myeloperoxidase, *P* Perinuclear, *PR3* Proteinase 3, *C* Cytoplasmic, *BVAS* Birmingham vasculitis activity score, *FFS* Five-factor score, *ESR* Erythrocyte sedimentation rate, *CRP* C-reactive protein, *GA* Glycated albumin, *HbA1c* Haemoglobin A1c, *ESRD* End-stage renal disease

### Medications and poor outcomes during follow-up

Data on medications and poor outcomes of AAV during follow-up were also collected, as described in Table 1. The medications included immunosuppressive drugs administered during follow-up. Poor outcomes of AAV included all-cause mortality, relapse, and the development of end-stage renal disease (ESRD). All-cause mortality was defined as death due to any aetiology, and relapse was defined as an increase in disease activity after the achievement of remission. ESRD was defined as a medical condition requiring renal replacement therapy. The follow-up duration based on mortality was defined as the period from AAV diagnosis to death in deceased patients. The follow-up durations based on relapse and ESRD were defined as the periods from AAV diagnosis to the time of relapse and the initiation of renal replacement therapy, respectively. In patients without poor outcomes, the follow-up duration was defined as the period from AAV diagnosis to the last visit in surviving patients.

### Statistical analyses

All statistical analyses were performed using IBM Statistical Product and Service Solutions Statistics for Windows, version 25 (IBM Corp., Armonk, NY, USA). Continuous variables are expressed as medians with interquartile ranges, whereas categorical variables are expressed as numbers (percentages). Significant differences between the two categorical variables were analysed using the Chi-square and Fisher’s exact tests. The Mann–Whitney U test was used to compare significant differences between two continuous variables. The correlation coefficient (r) between the two variables was obtained using either the Pearson correlation analysis or the univariable linear regression analysis. The optimal cut-off was extrapolated by performing the receiver operator characteristic (ROC) curve analysis and one value having the maximised sum of sensitivity and specificity was selected. The relative risk (RR) of the cut-off for the high activity of AAV was analysed using contingency tables and the chi-square test. Comparison of the cumulative survival rates between the two groups was performed using the Kaplan–Meier survival analysis with the log-rank test. *P*-values less than 0.05 were considered statistically significant.

## Results

### Comparison of inflammation-related biomarkers and fasting glucose between AAV patients with DM and those without DM (*N* = 105)

AAV patients with DM exhibited significantly higher GA (15.2 vs.14.0%), HbA1c (6.3 vs. 5.6%), and fasting glucose (106.0 mg/dL vs. 90.0 mg/dL) levels than those without DM, whereas, no significant difference in GA/HbA1c (2.9 vs. 2.4) values was observed between the two groups (See Supplementary Figure S1, Additional File 1).

### Characteristics of AAV patients without DM (*N* = 76)

The median age of AAV patients was 61 years, and 31 patients were male. Of the 76 AAV patients, 42 were classified as MPA; 18 as GPA; and 16 as EGPA. The median BVAS and FFS were 6.0 and 1.0. The median GA, HbA1c, and GA/HbA1c were 13.9%, 5.6%, and 2.4, respectively. Glucocorticoids were administered to 97.4% of patients. As induction therapy, cyclophosphamide and rituximab were administered to 64.5% and 23.7% of patients, respectively. During a median follow-up duration based on all-cause mortality of 28.7 months, five patients died. Meanwhile, 23 patients experienced relapse and eight patients encountered progression to ESRD (Table 1).

### Correlation of inflammation-related biomarkers with variables at diagnosis

GA was positively correlated with FFS (*r* = 0.282), BVAS assigned to renal manifestation (*r* = 0.315), and blood urea nitrogen (*r* = 0.382), whereas it was negatively correlated with haemoglobin (*r* = -0.345). GA/HbA1c, it was positively correlated with BVAS assigned to renal manifestation (*r* = 0.310), blood urea nitrogen (*r* = 0.487), and serum creatinine (*r* = 0.277), whereas inversely correlated with haemoglobin (*r* = -0.413). However, neither GA nor GA/HbA1c was significantly correlated with BVAS. In terms of HbA1c, there was no significant correlation with other variables in this study (Table 2).

**Table 2:** Correlation of inflammation-related biomarkers with variables at diagnosis in AAV patients (*N* = 76)

Variables at diagnosis	Correlation Coefficient (*r*, (*P*-value))		
***New biomarkers***	***GA***	***HbA1c***	***GA/HbA1c***
**AAV-specific indices**			
BVAS	0.116 (0.318)	0.040 (0.732)	0.090 (0.437)
FFS	0.282 (0.019)	0.069 (0.573)	0.209 (0.085)
**Clinical manifestations (Score assigned to each manifestation)**			
General manifestations	0.164 (0.156)	0.118 (0.308)	0.085 (0.463)
Cutaneous manifestations	-0.178 (0.123)	0.014 (0.907)	-0.159 (0.170)
Mucous and ocular manifestations	-0.051 (0.660)	0.046 (0.693)	-0.072 (0.536)
Otorhinolaryngologic manifestations	-0.082 (0.483)	0.033 (0.777)	-0.091 (0.434)
Pulmonary manifestations	-0.142 (0.221)	-0.118 (0.311)	-0.058 (0.618)
Cardiovascular manifestations	0.050 (0.669)	0.011 (0.923)	0.035 (0.767)
Gastrointestinal manifestations	N/A	N/A	N/A
Renal manifestations	0.315 (0.006)	-0.024 (0.834)	0.310 (0.006)
Nervous systemic manifestations	-0.088 (0.448)	0.182 (0.116)	-0.190 (0.101)
**Acute phase reactants**			
ESR (mm/hr)	0.146 (0.210)	0.048 (0.684)	0.094 (0.420)
CRP (mg/L)	0.124 (0.302)	-0.016 (0.894)	0.133 (0.270)
**Laboratory results**			
White blood cell count (/mm^3^)	-0.221 (0.055)	0.013 (0.914)	-0.226 (0.050)
Haemoglobin (g/dL)	-0.345 (0.002)	0.116 (0.317)	-0.416 (< 0.001)
Platelet count (× 1000/mm^3^)	0.031 (0.790)	-0.113 (0.331)	0.105 (0.365)
Blood urea nitrogen (mg/dL)	0.382 (0.001)	-0.158 (0.175)	0.487 (< 0.001)
Serum creatinine (mg/dL)	0.188 (0.107)	-0.128 (0.273)	0.277 (0.016)
Total protein (g/dL)	0.119 (0.309)	-0.024 (0.838)	0.094 (0.424)
Serum albumin (g/dL)	0.077 (0.512)	-0.067 (0.567)	0.103 (0.381)
Random urine protein/creatinine ratio	0.095 (0.439)	-0.076 (0.536)	0.152 (0.217)
Red blood cells per high power field	0.055 (0.646)	-0.043 (0.720)	0.067 (0.517)

*AAV* ANCA-associated vasculitis, *ANCA* Antineutrophil cytoplasmic antibody, *GA* Glycated albumin, *HbA1c* Haemoglobin A1c, *BVAS* Birmingham vasculitis activity score, *FFS* Five-factor score, *ESR* Erythrocyte sedimentation rate, *CRP* C-reactive protein

### Comparison of inflammation-related biomarkers between AAV patients with each poor outcome and those without

There were no significant differences in GA, HbA1c, and GA/HbA1c between surviving and deceased patients and between AAV patients with relapse and those without. However, AAV patients with ESRD exhibited significantly higher GA than those without ESRD (15.8 vs. 13.6%, *P* = 0.019). In addition, AAV patients with ESRD had higher GA/HbA1c than those without ESRD, but the difference was not statistically significant (2.7 vs. 2.4, *P* = 0.074). However, HbA1c was not significantly different between ESRD patients and non-ESRD patients (Table 3). No significant differences were found in the medications administered during follow-up between AAV patients with ESRD and those without (See Supplementary Table S1, Additional File 2).

**Table 3:** Comparison of inflammation-related biomarkers at diagnosis between AAV patients with each poor outcome and those without

**Variables**	**Surviving patients (*N* = 71)**	**Deceased patients (*N* = 5)**	***P*-value**
GA (%)	13.8 (2.7)	14.3 (4.9)	0.933
HbA1c (%)	5.6 (0.7)	5.3 (1.6)	0.339
GA/HbA1c	2.4 (0.5)	2.5 (0.3)	0.530
**Variables**	**Patients without relapse (*N* = 53)**	**Patients with relapse (*N* = 23)**	***P*-value**
GA (%)	14.1 (2.8)	13.3 (2.5)	0.246
HbA1c (%)	5.6 (0.8)	5.5 (0.5)	0.139
GA/HbA1c	2.4 (0.5)	2.4 (0.5)	0.968
**Variables**	**Patients without ESRD (*N* = 68)**	**Patients with ESRD (*N* = 8)**	***P*-value**
GA (%)	13.6 (2.4)	15.8 (2.1)	0.019
HbA1c (%)	5.6 (0.7)	5.6 (1.5)	0.766
GA/HbA1c	2.4 (0.5)	2.7 (0.8)	0.074

Values are expressed as a median (interquartile range, IQR)

*ANCA* Antineutrophil cytoplasmic antibody, *AAV* ANCA-associated vasculitis, *GA* Glycated albumin, *HbA1c* Haemoglobin A1c, *ESRD* End-stage renal disease

### Optimal cut-offs of GA and GA/HbA1c for the prediction of ESRD development

**Fig. 2:**
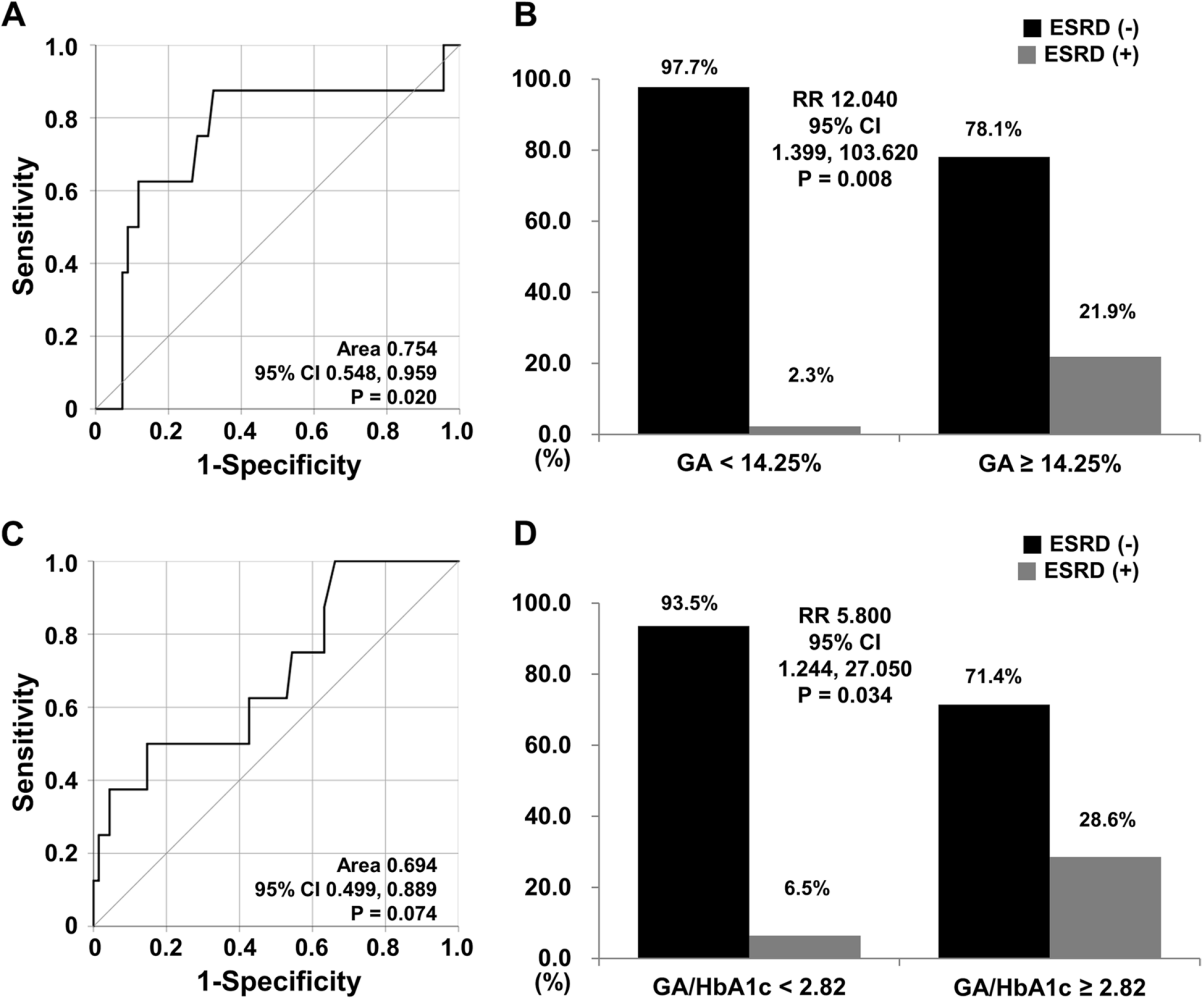
Cut-offs of GA and GA/HbA1c at diagnosis for ESRD and relative risks. **A** when the optimal cut-off of GA at diagnosis for ESRD development was set at GA ≥ 14.25%, the sensitivity and the specificity were 87.5% and 67.6%, respectively; **B** AAV patients with GA ≥ 14.25% had a significantly higher risk for ESRD development than those without (RR 12.040); **C** when the optimal cut-off of GA/HbA1c at diagnosis for ESRD development was set at GA/HbA1c ≥ 2.82, the sensitivity and the specificity were 50.0 and 85.3%, respectively; D) AAV patients with GA/HbA1c ≥ 2.82 showed a significantly higher risk for ESRD development than those without (RR 5.800). GA: glycated albumin; ESRD: end-stage renal disease; RR: relative risk; HbA1c: glycated haemoglobin

Since HbA1c was not correlated with any variable at initial diagnosis, including BVAS assigned to renal manifestation, and kidney-related parameters. Therefore, only cut-offs of GA and GA/HbA1c were calculated. When the optimal cut-off of GA at diagnosis for ESRD development was set at GA ≥ 14.25%, the sensitivity and the specificity were 87.5% and 67.6%, respectively (area under the curve 0.754, 95% confidence interval [CI] 0.548, 0.959) (Fig. 2A). When AAV patients were classified into two groups based on the cut-off of GA ≥ 14.25%, AAV patients with GA ≥ 14.25% had a significantly higher risk for ESRD development than those with GA < 14.25% (RR 12.040, 95% CI 1.399, 103.620) (Fig. 2B).

Contrarily, when the optimal cut-off of GA/HbA1c at diagnosis for ESRD development was set at GA/HbA1c ≥ 2.82, the sensitivity and the specificity were 50.0% and 85.3%, respectively (area under the curve 0.694, 95% CI 0.499, 0.889) (Fig. 2C). When AAV patients were divided into two groups based on the cut-off of GA/HbA1c ≥ 2.82, AAV patients with GA/HbA1c ≥ 2.82 showed a significantly higher risk for ESRD development than those with GA/HbA1c < 2.82 (RR 5.800, 95% CI 1.244, 27.050) (Fig. 2D).

### Comparison of the cumulative ESRD-free survival rates

**Fig. 3:**
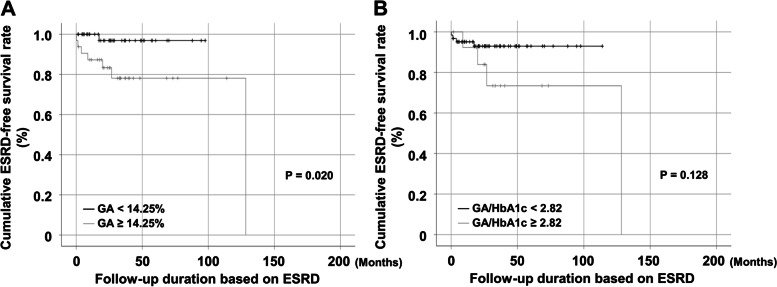
Comparison of the cumulative ESRD-free survival rates. **A** AAV patients with GA ≥ 14.25% exhibited significantly lower cumulative ESRD-free survival rates than those without; **B** the cumulative ESRD-free survival rates did not differ between AAV patients with GA/HbA1c ≥ 2.82 and those without. ESRD: end-stage renal disease; GA: glycated albumin; HbA1c: glycated haemoglobin

The cumulative ESRD-free survival rates were compared between AAV patients with GA ≥ 14.25% and those without or those with GA/HbA1c ≥ 2.82 and those without. AAV patients with GA ≥ 14.25% exhibited significantly lower cumulative ESRD-free survival rates than those with GA < 14.25% (Fig. 3A). However, the cumulative ESRD-free survival rates did not differ between AAV patients with GA/HbA1c ≥ 2.82 and those with GA/HbA1c < 2.82 (Fig. 3B).

## Discussion

In this study, GA/HbA1c was selected for two reasons: First, it was previously reported that GA/HbA1c could be used as a factor reflecting long-term glycaemic control along with GA and HbA1c [19]. Second, although GA/HbA1c did not show a significant difference between DM and non-DM patients, theoretically, elevated GA at diagnosis could reflect the extent of inflammation without confounding effects of hyperglycaemia in AAV patients. GA and GA/HbA1c were selected as biomarkers instead of HbA1c since it showed no significant correlation with any clinical or laboratory variable in the correlation analysis (Table 2). We first demonstrated that GA at diagnosis could reflect BVAS assigned to renal manifestation of AAV, and predict ESRD development during follow-up. In addition, GA/HbA1c also showed a pattern similar to that of GA, but the clinical significance was not as high as GA. It is important to highlight that GA levels may be influenced by decreased liver and renal function. GA levels are usually low in patients with decreased liver function. It seems that liver disease had the least effect on GA/HbA1c compared to GA and HbA1c [20, 21]. In addition, GA levels are not affected by non-nephrotic range of proteinuria in CKD patients. However, in CKD patients with nephrotic range of proteinuria, GA level is decreased independent of glycaemic status [22]. Therefore, it is important to interpret GA with caution in AAV patients with abnormal liver function of nephrotic range of proteinuria in CKD patients. In general, GA may be considered as a more useful biomarker that could reflect inflammation that could lead to renal failure in AAV patients.

It is unclear whether GA plays a causal role in the inflammatory process or is a mere consequence of the cascade. Advanced glycation end (AGE) products, including GA, which are mainly formed in a hyperglycaemic state, can be also found under inflammatory conditions [23, 24]. The receptor for AGEs (RAGE) is a pattern-recognition receptor, and once its ligands, and GA bind to RAGE, various intracellular signalling cascades are initiated [25, 26]. GA can upregulate the gene expression of monocyte chemoattractant protein-1, IL-6, and IL-8 via nuclear factor-kappa B signalling, and it can also enhance the expression of c-fos and c-jun via extracellular signal-regulated kinases, comparable with tumour necrosis factor-α, IL-1β or lipopolysaccharide [8, 26]. An increase in the production of GA due to systemic inflammation can further aggravate inflammation, in addition to the deterioration of glucose metabolism, resulting in an amplified vicious cycle. Although we could not definitively determine whether increased GA production might clinically exacerbate AAV activity in the present study, it can be hypothesised upon with reasonable confidence, as the confounding factor of DM was eliminated from this study. Moreover, elevated GA at diagnosis may reflect the extent of inflammation without the confounding effect of hyperglycaemia.

Contrary to the initial assumption, GA was not directly correlated with BVAS in this study. Given that BVAS is composed of nine systemic items, correlations with each systemic item were investigated, and it was confirmed that BVAS assigned to renal manifestation showed a significant correlation. In addition, there was a significant difference in GA between AAV patients with ESRD and those without ESRD, and GA was found to be a possible predictor renal failure leading to ESRD. A previous 5-year prospective population-based study reported that the baseline value of GA independently and significantly predicted renal dysfunction, along with age and uric acid [27]. Therefore, in the clinical setting, GA, as a biomarker, can reflect the cross-sectional renal manifestation and predict progression to ESRD in AAV rather than directly reflecting AAV activity.

A question arises as to why HbA1c, also a glycated protein, did not show a significant correlation with BVAS assigned to renal manifestation. In a previous study, it was reported that HbA1c may not be a reliable marker for glycaemic state in cases involving renal comorbidities, haemoglobinopathies, and pregnancy, and further, GA could overcome the limitations of HbA1c in these medical conditions [28]. In this study, AAV activity showed an inverse correlation with haemoglobin. Therefore, the association between the degree of inflammation represented by BVAS and HbA1c may not be significant because the enhanced activity of AAV may exacerbate anaemia due to insufficient production of erythropoietin [28]. Moreover, renal manifestation itself could reduce the reliability of HbA1c, leading to discordance in the proportionality of the AAV-related inflammatory burden between HbA1c and GA.

In this study, both GA and GA/HbA1c were demonstrated to have potential as biomarkers for assessing the cross-sectional extent of renal involvement in AAV and predicting the progression to ESRD. GA showed significant correlations with renal manifestation, exhibiting a difference between AAV patients with ESRD and those without. Using an optimal cut-off, GA could predict the relative risk of ESRD as well as ESRD occurrence, with the Kaplan–Meier survival analysis. However, GA/HbA1c did not show a significant difference based on the presence or absence of ESRD. Its predictive potential for ESRD occurrence using the Kaplan–Meier survival analysis was not significant. Therefore, we suggest GA rather than GA/HbA1c as a novel biomarker for renal manifestation in AAV patients.

Recently, albumin-adjusted GA (the ratio of GA to serum albumin level) was suggested as a new indicator for glycaemic control [29]. We know that inflammatory burden initiates and accelerates the production of GA, along with the hyperglycaemic state, but serum albumin falls in an inflammatory state. Therefore, we can expect that albumin-adjusted GA would increase as AAV activity rises, and predict poor outcomes better than GA. First, in terms of the cross-sectional BVAS, we conducted another correlation analysis and found that albumin-adjusted GA showed significant correlation with the cross-sectional BVAS (*r* = 0.453, *P* < 0.001) and BVAS assigned to renal manifestation (*r* = 0.501, *P* < 0.001). Therefore, albumin-adjusted GA could be used as a biomarker to directly reflect the cross-sectional both BVAS and BVAS assigned to renal manifestation in AAV patients.

Second, in terms of ESRD occurrence, AAV patients with ESRD exhibited a higher median albumin-adjusted GA than those without ESRD (3.7 vs. 3.4, *P* = 0.041). However, in the ROC curve analysis to obtain an optimal cut-off, albumin-adjusted GA (area under the curve 0.752, 95% CI 0.547, 0.957) exhibited a lower area than GA (area under the curve 0.722, 95% CI 0.563, 0.881) (See Supplementary Figure S2, Additional File 3). In addition, the optimal cut-off of albumin-adjusted GA for ESRD occurrence was set at 3.42 with the sensitivity and the specificity of 87.5% and 58.2%, respectively. However, the relative risk of albumin-adjusted GA ≥ 3.42 for ESRD occurrence was lower than that of GA ≥ 14.25% (9.172 vs. 12.040). Although albumin-adjusted GA ≥ 3.42 could significantly predict ESRD occurrence during the follow-up duration based on ESRD, the statistical significance of albumin-adjusted GA did not surpass that of GA (*P* = 0.046 vs. *P* = 0.020) (See Supplementary Figure S3, Additional File 4). Therefore, GA could predict ESRD occurrence during follow-up better than albumin-adjusted GA. Based on these results, we suggest that GA, rather than albumin-adjusted GA, is more clinically helpful in predicting ESRD occurrence.

There are traditional and conventional risk factors that predicts the progression to ESRD in general population. Serum creatinine and age are well known risk factor for ESRD. We conducted a Cox hazard model analysis to evaluation the predictive ability of several laboratory variables and patient characteristics including age and gender. In univariate analysis, BVAS, white blood cell count, haemoglobin, serum creatinine and GA ≥ 14.25% were statistically significant. In multivariate analysis, only serum creatinine was proven to be a significant predictor for ESRD in AAV patients without DM (HR 1.323, 95% CI 1.019, 1.717, *P* = 0.036) (See Supplementary Table S2, Additional File 5). The predictive ability of GA could not surpass that of serum creatinine. However, we believe that GA could give additional clinical information to physicians for predicting ESRD in AAV patients without DM. With a multi-centric and prospective future study with a large number of patients, it will provide more dynamic and clearer information on the clinical usefulness of GA in predicting ESRD in AAV patients and will validate the results of our study further.

This study has several limitations. First, the retrospective study design did not allow for the serial collection of both GA and HbA1c results in non-DM patients with AAV. Second, the number of patients was not large enough to generalise the results of this study for application in all patients with AAV. Third, GA has been reported to be capable of predicting the development of DM in pre-diabetic or euglycemic patients [30]; however, we could not evaluate it because it was not easy to distinguish the causes of elevated glucose levels (isolated DM versus the drugs for AAV treatment, such as steroids and calcineurin inhibitors that can increase blood sugar. However, for the first time, we demonstrated the predictive capability of GA for the extent of renal involvement in AAV, and thus, our study has clinical significance as a pilot study. A future study with a larger number of patients and with serial results of both GA and HaA1c can validate our study findings and provide more information on the clinical role of GA in AAV.

In conclusion, GA at diagnosis can reflect BVAS assigned to renal manifestation of AAV and predict renal failure to progress to ESRD during follow-up better than HbA1c or GA/HbA1c in non-DM patients with AAV. Therefore, we expect that GA may be used as a biomarker for renal dysfunction and ESRD occurrence during follow-up in AAV patients.

## Supplementary Information

**Additional file 1: Supplementary Figure S1.** Comparison of inflammation-related biomarkers and fasting glucose between AAV patients with DM and those without DM. AAV patients with DM exhibited significantly higher GA, HbA1c, and fasting glucose levels than those without DM, whereas, no significant difference in GA/HbA1c values was observed between the two groups.

**Additional file 2: Supplementary Table S1.** Comparison of medications administered during follow-up between AAV patients with ESRD and those without.

**Additional file 3: Supplementary Figure S2.** Comparison of area under the curve in the ROC curve for ESRD between GA and albumin-adjusted GA. Regarding the development of ESRD in AAV patients, albumin-adjusted GA (area under the curve 0.752) exhibited a lower area than GA (area under the curve 0.722) for ESRD in AAV patients.

**Additional file 4: Supplementary Figure S3.** Comparison of the predictive potential for ESRD development between GA and albumin-adjusted GA. Although albumin-adjusted GA ≥ 3.42 could significantly predict ESRD occurrence during the follow-up duration based on ESRD, the statistical significance of albumin-adjusted GA did not surpass that of GA (P = 0.046 vs. P = 0.020) in AAV patients.

**Additional file 5: Supplementary Table S2.** Cox hazards model analysis of GA and other variables at diagnosis for ESRD during follow-up in AAV patients without DM.

## Data Availability

The datasets used and/or analysed during the current study are available from the corresponding author on reasonable request.

## Abbreviations

GA: Glycated albumin
HbA1c: Glycated haemoglobin
DM: Diabetes mellitus
ANCA: Antineutrophil cytoplasmic antibody
AAV: ANCA-associated vasculitis
BVAS: Birmingham vasculitis activity score
FFS: Five-factor score
ESRD: End-stage renal disease
IL: Interleukin
MPA: Microscopic polyangiitis
GPA: Granulomatosis with polyangiitis
EGPA: Eosinophilic GPA
P-ANCA: Perinuclear-ANCA
C-ANCA: Cytoplasmic-ANCA
MPO: Myeloperoxidase
PR3: Proteinase 3
ROC: Receiver operator characteristic
RR: Relative risk
CI: Confidence interval
SHAVE: Severance Hospital ANCA-associated VasculitidEs
IRB: Institutional Review Board
AGE: Advanced glycation end
RAGE: Receptor for AGEs
