# Supplementary material for: Serum glycated albumin as a predictive biomarker for renal involvement of antineutrophil cytoplasmic antibody-associated vasculitis in non-diabetic patients
Source: BMC Nephrol. 2022 Aug 18;23:288. doi: 10.1186/s12882-022-02913-5 (PMC9389827; doi:10.1186/s12882-022-02913-5)
Supplement: Supplementary file 1 — Additional file 1: Supplementary Figure S1. Comparison of inflammation-related biomarkers and fasting glucose between AAV patients with DM and those without DM. AAV patients with DM exhibited significantly higher GA, HbA1c, and fasting glucose levels than those without DM, whereas, no significant difference in GA/HbA1c values was observed between the two groups. [file 12882_2022_2913_MOESM1_ESM.docx]

**Additional File 1: Supplementary Figure S1. Comparison of inflammation-related biomarkers and fasting glucose between AAV patients with DM and those without DM. AAV patients with DM exhibited significantly higher GA, HbA1c, and fasting glucose levels than those without DM, whereas, no significant difference in GA/HbA1c values was observed between the two groups.**


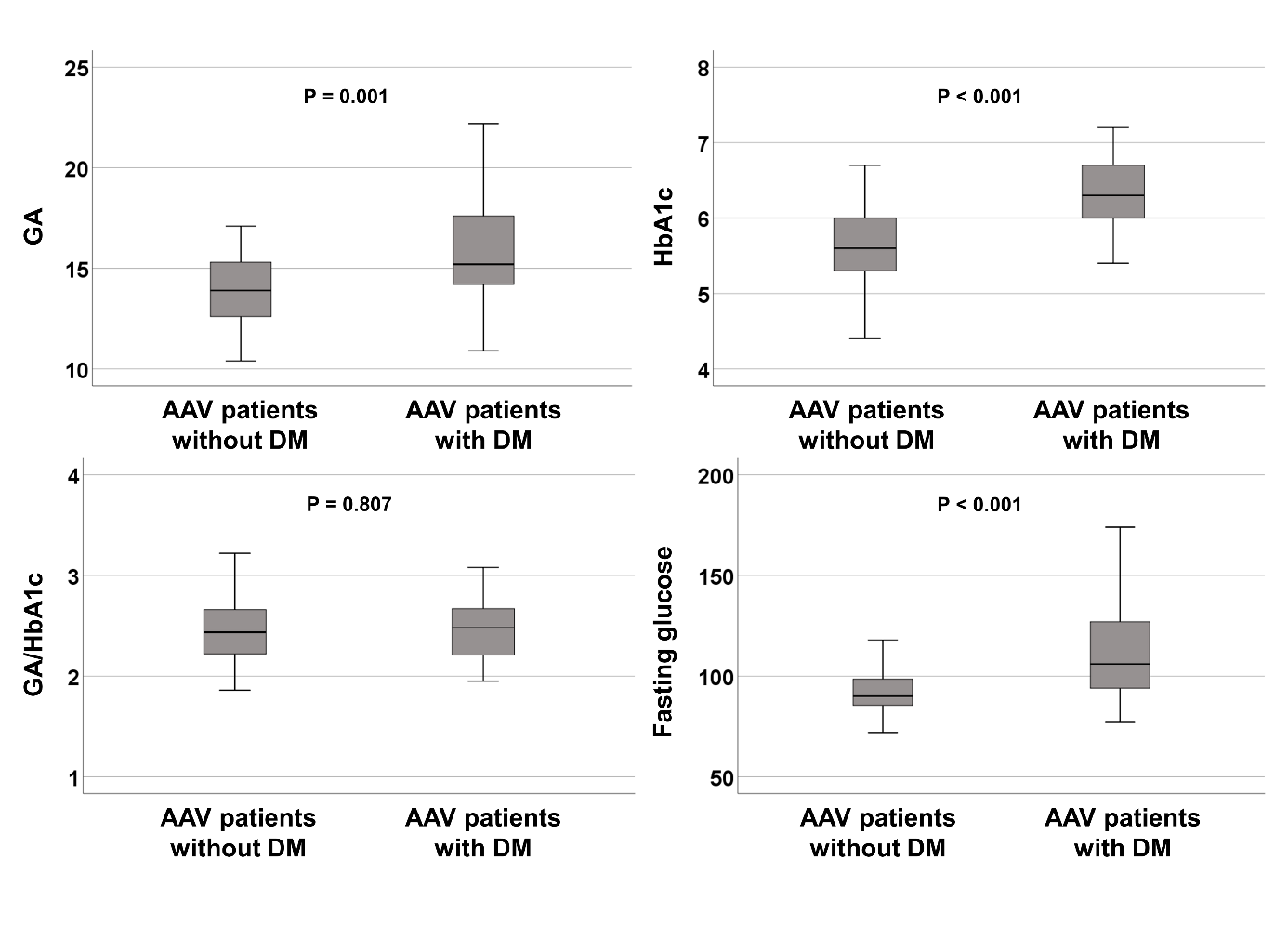
AAV: ANCA-associated vasculitis; ANCA: antineutrophil cytoplasmic antibody; DM: diabetes mellitus; GA: glycated albumin; HbA1c: glycated haemoglobin.
