# Supplementary material for: Serum glycated albumin as a predictive biomarker for renal involvement of antineutrophil cytoplasmic antibody-associated vasculitis in non-diabetic patients
Source: BMC Nephrol. 2022 Aug 18;23:288. doi: 10.1186/s12882-022-02913-5 (PMC9389827; doi:10.1186/s12882-022-02913-5)
Supplement: Supplementary file 2 — Additional file 2: Supplementary Table S1. Comparison of medications administered during follow-up between AAV patients with ESRD and those without. [file 12882_2022_2913_MOESM2_ESM.docx]

**Additional File 2: Supplementary Table S1. Comparison of medications administered during follow-up between AAV patients with ESRD and those without.**

| **Variables (N, (%))** | **Patients without ESRD**  **(N = 68)** | **Patients with**  **ESRD**  **(N = 8)** | **P-value** |
| --- | --- | --- | --- |
| Glucocorticoids | 66 (97.1) | 8 (100) | 0.623 |
| Cyclophosphamide | 43 (63.2) | 6 (75.0) | 0.511 |
| Rituximab | 15 (22.1) | 3 (37.5) | 0.385 |
| Mycophenolate mofetil | 10 (14.7) | 2 (25.0) | 0.605 |
| Azathioprine | 49 (72.1) | 3 (37.5) | 0.100 |
| Tacrolimus | 4 (5.9) | 0 (0) | 1.000 |
| Methotrexate | 8 (11.8) | 0 (0) | 0.589 |
| Plasma exchange | 7 (10.3) | 3 (37.5) | 0.066 |

Values are expressed as N (%).

ANCA: antineutrophil cytoplasmic antibody; AAV: ANCA-associated vasculitis; ESRD: end-stage renal disease.
