# Supplementary material for: Serum glycated albumin as a predictive biomarker for renal involvement of antineutrophil cytoplasmic antibody-associated vasculitis in non-diabetic patients
Source: BMC Nephrol. 2022 Aug 18;23:288. doi: 10.1186/s12882-022-02913-5 (PMC9389827; doi:10.1186/s12882-022-02913-5)
Supplement: Supplementary file 3 — Additional file 3: Supplementary Figure S2. Comparison of area under the curve in the ROC curve for ESRD between GA and albumin-adjusted GA. Regarding the development of ESRD in AAV patients, albumin-adjusted GA (area under the curve 0.752) exhibited a lower area than GA (area under the curve 0.722) for ESRD in AAV patients. [file 12882_2022_2913_MOESM3_ESM.docx]

**Additional File 3: Supplementary Figure S2. Comparison of area under the curve in the ROC curve for ESRD between GA and albumin-adjusted GA. Regarding the development of ESRD in AAV patients, albumin-adjusted GA (area under the curve 0.752) exhibited a lower area than GA (area under the curve 0.722) for ESRD in AAV patients.**


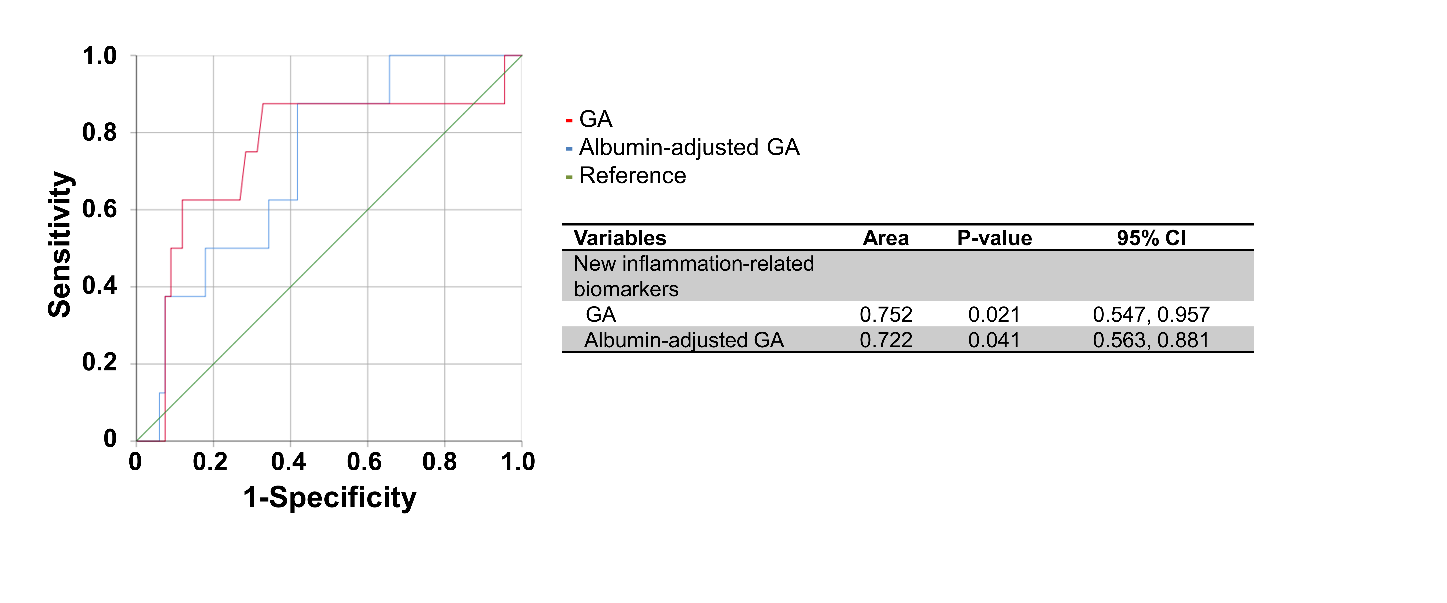
ESRD: end-stage renal disease; AAV: ANCA-associated vasculitis; ANCA: antineutrophil cytoplasmic antibody; GA: glycated albumin.
