# Supplementary material for: Serum glycated albumin as a predictive biomarker for renal involvement of antineutrophil cytoplasmic antibody-associated vasculitis in non-diabetic patients
Source: BMC Nephrol. 2022 Aug 18;23:288. doi: 10.1186/s12882-022-02913-5 (PMC9389827; doi:10.1186/s12882-022-02913-5)
Supplement: Supplementary file 4 — Additional file 4: Supplementary Figure S3. Comparison of the predictive potential for ESRD development between GA and albumin-adjusted GA. Although albumin-adjusted GA ≥ 3.42 could significantly predict ESRD occurrence during the follow-up duration based on ESRD, the statistical significance of albumin-adjusted GA did not surpass that of GA (P = 0.046 vs. P = 0.020) in AAV patients. [file 12882_2022_2913_MOESM4_ESM.docx]

**Additional File 4: Supplementary Figure S3. Comparison of the predictive potential for ESRD development between GA and albumin-adjusted GA. Although albumin-adjusted GA ≥ 3.42 could significantly predict ESRD occurrence during the follow-up duration based on ESRD, the statistical significance of albumin-adjusted GA did not surpass that of GA (P = 0.046 vs. P = 0.020) in AAV patients.**


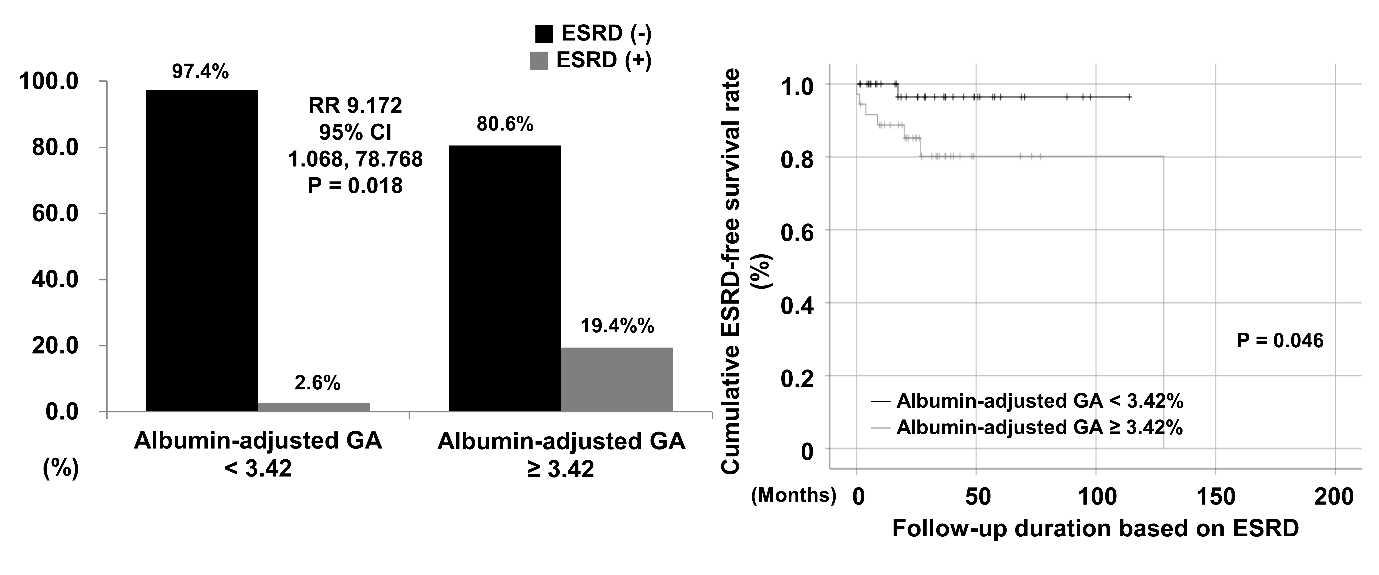
GA: glycated albumin; ESRD: end-stage renal disease; AAV: ANCA-associated vasculitis; ANCA: antineutrophil cytoplasmic antibody.
