# Supplementary material for: Serum glycated albumin as a predictive biomarker for renal involvement of antineutrophil cytoplasmic antibody-associated vasculitis in non-diabetic patients
Source: BMC Nephrol. 2022 Aug 18;23:288. doi: 10.1186/s12882-022-02913-5 (PMC9389827; doi:10.1186/s12882-022-02913-5)
Supplement: Supplementary file 5 — Additional file 5: Supplementary Table S2. Cox hazards model analysis of GA and other variables at diagnosis for ESRD during follow-up in AAV patients without DM. [file 12882_2022_2913_MOESM5_ESM.docx]

**Additional File 5: Supplementary Table S2. Cox hazards model analysis of GA and other variables at diagnosis for ESRD during follow-up in AAV patients without DM.**

| **Variables** | **Univariable** | | |  | **Multivariable (HSI)** | | |
| --- | --- | --- | --- | --- | --- | --- | --- |
|  | **HR** | **95% CI** | **P value** |  | **HR** | **95% CI** | **P value** |
| Age (years) | 1.037 | 0.971, 1.106 | 0.278 |  |  |  |  |
| Male sex | 1.313 | 0.289, 5.959 | 0.724 |  |  |  |  |
| MPO-ANCA (or P-ANCA) positivity | 1.024 | 0.229, 4.581 | 0.975 |  |  |  |  |
| PR3-ANCA (or C-ANCA) positivity | 1.241 | 0.149, 10.310 | 0.842 |  |  |  |  |
| BVAS | 1.233 | 1.083, 1.404 | 0.002 |  | 1.117 | 0.931, 1.339 | 0.234 |
| FFS | 1.893 | 0.805, 4.452 | 0.144 |  |  |  |  |
| ESR (mm/hr) | 1.008 | 0.990, 1.027 | 0.367 |  |  |  |  |
| CRP (mg/L) | 1.012 | 0.992, 1.032 | 0.250 |  |  |  |  |
| White blood cell count (/mm^3^) | 1.174 | 1.000, 1.379 | 0.050 |  | 1.151 | 0.916, 1.447 | 0.227 |
| Haemoglobin (g/dL) | 0.648 | 0.430, 0.978 | 0.039 |  | 0.882 | 0.554, 1.403 | 0.595 |
| Platelet count (× 1000/mm^3^) | 0.995 | 0.986, 1.005 | 0.315 |  |  |  |  |
| Serum creatinine (mg/dL) | 1.366 | 1.146, 1.627 | <0.001 |  | 1.323 | 1.019, 1.717 | 0.036 |
| Total protein (g/dL) | 0.978 | 0.309, 3.099 | 0.970 |  |  |  |  |
| Serum albumin (g/dL) | 0.398 | 1.110, 1.443 | 0.161 |  |  |  |  |
| GA ≥ 14.25% | 8.241 | 0.992, 68.470 | 0.051 |  | 2.528 | 0.220, 29.077 | 0.457 |

GA: glycated albumin; ESRD: end-stage renal disease; AAV: ANCA-associated vasculitis; ANCA: antineutrophil cytoplasmic antibody; DM: diabetes mellitus; MPO: myeloperoxidase; P: perinuclear; PR3: proteinase 3; C: cytoplasmic; BVAS: Birmingham vasculitis activity score; FFS: five-factor score; ESR: erythrocyte sedimentation rate; CRP: C-reactive protein.
